# Luteolin-mediated Kv1.3 K^+^ channel inhibition augments BCG vaccine efficacy against tuberculosis by promoting central memory T cell responses in mice

**DOI:** 10.1371/journal.ppat.1008887

**Published:** 2020-09-21

**Authors:** Dhiraj Kumar Singh, Ved Prakash Dwivedi, Shashi Prakash Singh, Anjna Kumari, Saurabh Kumar Sharma, Anand Ranganathan, Luc Van Kaer, Gobardhan Das

**Affiliations:** 1 Special Centre for Molecular Medicine (SCMM), Jawaharlal Nehru University, New Delhi, India; 2 International Centre for Genetic Engineering and Biotechnology, New Delhi, India; 3 Southwest National Primate Research Center, Texas Biomedical Research Institute, San Antonio, Texas, United States of America; 4 School of Computer & Systems Sciences, Jawaharlal Nehru University, New Delhi, India; 5 Department of Pathology, Microbiology and Immunology, Vanderbilt University School of Medicine, Nashville, Tennessee, United States of America; Portland VA Medical Center, Oregon Health and Science University, UNITED STATES

## Abstract

Despite the availability of multiple antibiotics, tuberculosis (TB) remains a major health problem worldwide, with one third of the population latently infected and ~2 million deaths annually. The only available vaccine for TB, Bacillus Calmette Guérin (BCG), is ineffective against adult pulmonary TB. Therefore, alternate strategies that enhance vaccine efficacy are urgently needed. Vaccine efficacy and long-term immune memory are critically dependent on central memory T (T_CM_) cells, whereas effector memory T (T_EM_) cells are important for clearing acute infections. Recently, it has been shown that inhibition of the Kv1.3 K^+^ ion channel, which is predominantly expressed on T_EM_ but not T_CM_ cells, profoundly enhances T_CM_ cell differentiation. We exploited this phenomenon to improve T_CM_:T_EM_ cell ratios and protective immunity against *Mycobacterium tuberculosis* infection in response to BCG vaccination of mice. We demonstrate that luteolin, a plant-derived Kv1.3 K^+^ channel inhibitor, profoundly promotes T_CM_ cells by selectively inhibiting T_EM_ cells, and significantly enhances BCG vaccine efficacy. Thus, addition of luteolin to BCG vaccination may provide a sustainable means to improve vaccine efficacy by boosting host immunity via modulation of memory T cell differentiation.

## Author summary

Bacillus Calmette Guérin (BCG) is not effective against adult pulmonary tuberculosis (TB). Inhibition of the Kv1.3 K^+^ ion channel by the antibiotic clofazimine has been shown to enhance BCG-induced immunity. However, clofazimine has limited efficacy and is associated with substantial side effects in treated patients. Therefore, we explored alternatives to clofazimine. Luteolin is a plant-based flavonoid that inhibits Kv1.3. We show that administration of luteolin during BCG vaccination enhances antigen-specific immunity by promoting the T central memory (T_CM_) cell pool, which is critically important for long term host protection. Consequently, luteolin-mediated immune modulation enhances vaccine efficacy. As luteolin is a biologically safe food supplement, it could be easily applied during vaccination.

## Introduction

Despite the availability of multiple effective antibiotics, tuberculosis (TB) has emerged as the greatest killer among all infectious diseases, with one third of the global population infected, and 10.4 million new cases and ~1.74 million deaths reported in 2016 [1]. Bacillus Calmette Guérin (BCG), the only usable TB vaccine currently available, is over a century old and fails to protect against adult pulmonary TB [2]. BCG elicits sufficient host protective T helper (Th) 1 (producers of IFN-γ) responses and exhibits efficacy against disseminated and meningeal TB in neonates. However, these cells become gradually exhausted and the host becomes again susceptible to *Mycobacterium tuberculosis* (*M*.*tb*) infection [3–5]. It is now increasingly clear that T effector memory (T_EM_) cells with a Th1 cytokine production profile provide protection against acute TB infection, whereas T central memory (T_CM_) cells provide vaccine efficacy by generating T_EM_ cells. Therefore, during the course of an ongoing infection, a wider pool of T_CM_ cells is desired to provide the host with a continuous supply of T_EM_ cells. The generation of T_EM_ cells is proportionate to bacterial burden and these cells rapidly produce copious amounts of IFN-γ, which promotes cellular immune responses and eliminates bacteria. T_EM_ cells, however, are terminally differentiated effector cells with low or no proliferative capacity [6–8]. Therefore, maintenance of long-term protective memory responses is thought to rely on T_CM_ cells with high proliferative capacity [9]. T_CM_ responses play a critical role in driving cell-mediated host immune responses and thereby the efficacy of TB vaccines [10,11]. Therefore, restoring host T_CM_ responses may enhance TB vaccine efficacy. Increasing the pool of T_CM_ (CD44^hi^CD62L^hi^ or CD44^hi^CCR7^hi^) cells by concomitant regulation of T_EM_ (CD44^hi^CD62L^lo^ or CD44^hi^CCR7^lo^) cells may be an effective strategy to develop long-lasting and robust recall responses.

In order to specifically modulate T_CM_ cells without altering T_EM_ cell responses, an intricate understanding of the differential physiology between these two T cell subsets is required. Kv1.3, a potassium ion channel, is predominantly expressed on T_EM_ cells and plays an important role in the maintenance and effector functions of these cells [12,13]. On the other hand, T_CM_ cells express higher levels of the KCa3.1 ion channel, which drives and maintains the activation of this memory T cell subset. In keeping with these findings, several independent studies have shown that knock-down of Kv1.3 results in differential modulation of T_CM_ vs. T_EM_ cells, to favor T_CM_ generation [13,14]. In fact, genetic deficiency of Kv1.3 has been shown to induce reversal of T_EM_ to T_CM_ cell ratios [13,14]. A recent study showed that inhibition of Kv1.3 K^+^ ion channels by the antibiotic clofazimine enhances the pool of T_CM_ cells, and these cells have the potential to continuously replace effector T cells at the site of infection, thereby improving host immunity [15]. Clofazimine, a well-known anti-leprotic drug, is reserved as a class 5 drug for TB and is currently only being used in long regimens to treat extremely drug-resistant (XDR) TB. The half-life of clofazimine in lungs is estimated at over 4 weeks, and this drug also accumulates in various organs [16]. Therefore, employing clofazimine during immunization of infants and adults is suboptimal.

In searching for a biologically safe alternative Kv1.3 inhibitor we explored 3,4,5,7-tetrahydroxyflavone, also known as luteolin, a plant-based flavonoid that inhibits Kv1.3 [17]. Luteolin has recently been employed as a food supplement and is considered safe for human use. It is a flavonoid found in many fruits, vegetables, and medicinal plants such as *Reseda luteola* L., *Achillea millefolium* L. and many others. Luteolin-rich herbal extracts have been used for a long time as traditional herbal remedies [18,19]. We observed that luteolin had mild bactericidal activity in vitro (S1 Fig), as reported [20]. Moreover, we also found that luteolin treatment significantly activated macrophages, as evidenced by increased expression of co-stimulatory molecules and improved bactericidal activity (S2A & S2B Fig). Taken together, these findings suggested that luteolin might have potent immunomodulatory effects, which along with selective enrichment of the T_CM_ pool may be highly beneficial in combating TB. Therefore, we performed a proof of concept study to explore the effects of luteolin on the memory T cell responses after BCG immunization, using the intraperitoneal route of administration to maintain homogeneity in dosage and to achieve higher bioavailability than the oral route [19,20].

Our results demonstrate that mice receiving luteolin during BCG vaccination exhibit superior host protection against TB, which was associated with potent Th1 and Th17 responses, and enrichment of CD44^hi^CD62L^hi^ and CD44^hi^CCR7^hi^ T_CM_ cells in both CD4^+^ and CD8^+^ compartments. Taken together, our findings reveal that specific immunomodulation of memory T cells during vaccination can enhance the efficacy of BCG vaccination and improve long-term host immunity.

## Results

### Luteolin biases T cells towards a T_CM_ phenotype *in vitro*

**Fig 1:**
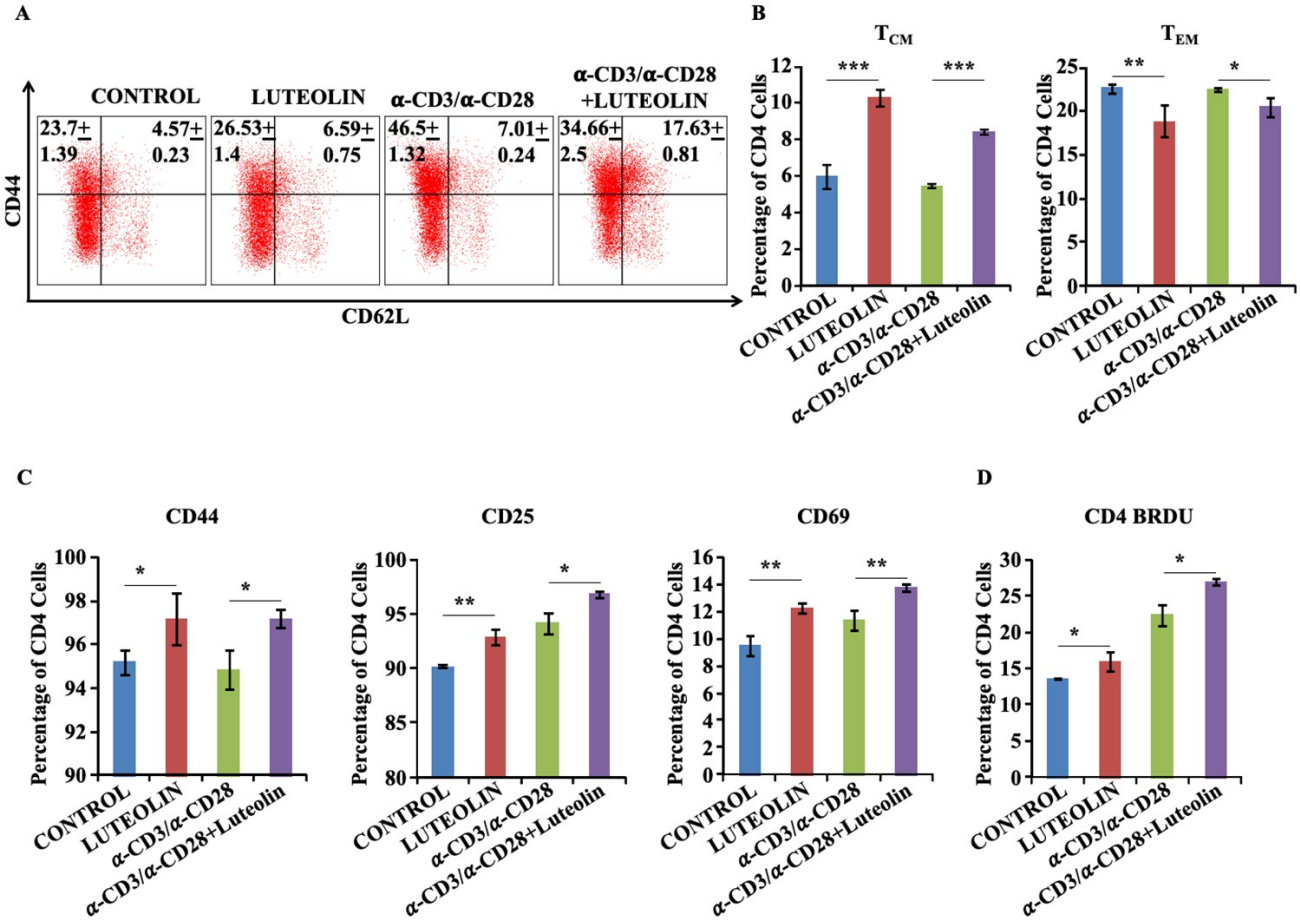
Luteolin biases T cells towards a T_CM_ phenotype after *in vitro* activation with plate bound α-CD3 & soluble α-CD28 antibodies. **(A&B)** Memory responses of CD4^+^ T cells *in vitro*. **(C)** T lymphocytes were activated *in vitro* with plate-bound α-CD3 & soluble α-CD28 antibodies in the presence or absence of luteolin and then stained with anti-CD4, -CD62L, -CD25, -CD44 and -CD69 mAbs. **(D)** T cell proliferation was monitored by BrdU incorporation post activation in vitro by staining with anti-CD4 and -BrdU antibodies. Data represented as the mean±STDEV values from data pooled from three experiments with five mice per experiment. Differences were considered significant at P<0.05 using one way ANOVA. *p<0.05, **p<0.005, ***p<0.0005.

Although the ability of luteolin to inhibit the Kv1.3 K^+^ ion channel was reported over a decade ago [17], its capacity to modulate memory T cell responses has not yet been explored. To specifically evaluate this phenomenon, we stimulated naïve T cells in the presence of luteolin and found that it was indeed capable of significantly promoting T_CM_ responses *in vitro* both with and without prior T cell activation by anti-CD3 and anti-CD28 mAbs (Fig 1A & 1B). In addition, luteolin also enhanced CD4^+^ T cell activation as evidenced by increased prevalence of CD44^+^, CD25^+^ and CD69^+^ cells (Fig 1C) and proliferation (Fig 1D) measured by BrdU incorporation assay. These data suggest that luteolin selectively induces the T_CM_ cell population, which is considered the main driver of recall responses. As per the recent literature, the first exposure to a pathogen generates a primary effector T cell repertoire which then clears the primary infection and contracts to give rise to a T_CM_ cell pool. On subsequent challenge a secondary response is generated by the rapid proliferation of T_CM_ cells which give rise to secondary effector T cells that drive clearance of the pathogen. Although effector T cells are the main drivers of pathogen clearance, their rapid clonal expansion during a secondary immune response is chiefly driven by the T_CM_ pool [21, 22].

### Luteolin induces sustained T cell immunity in lung and spleen of BCG-immunized mice challenged with *M*.*tb*

**Fig 2:**
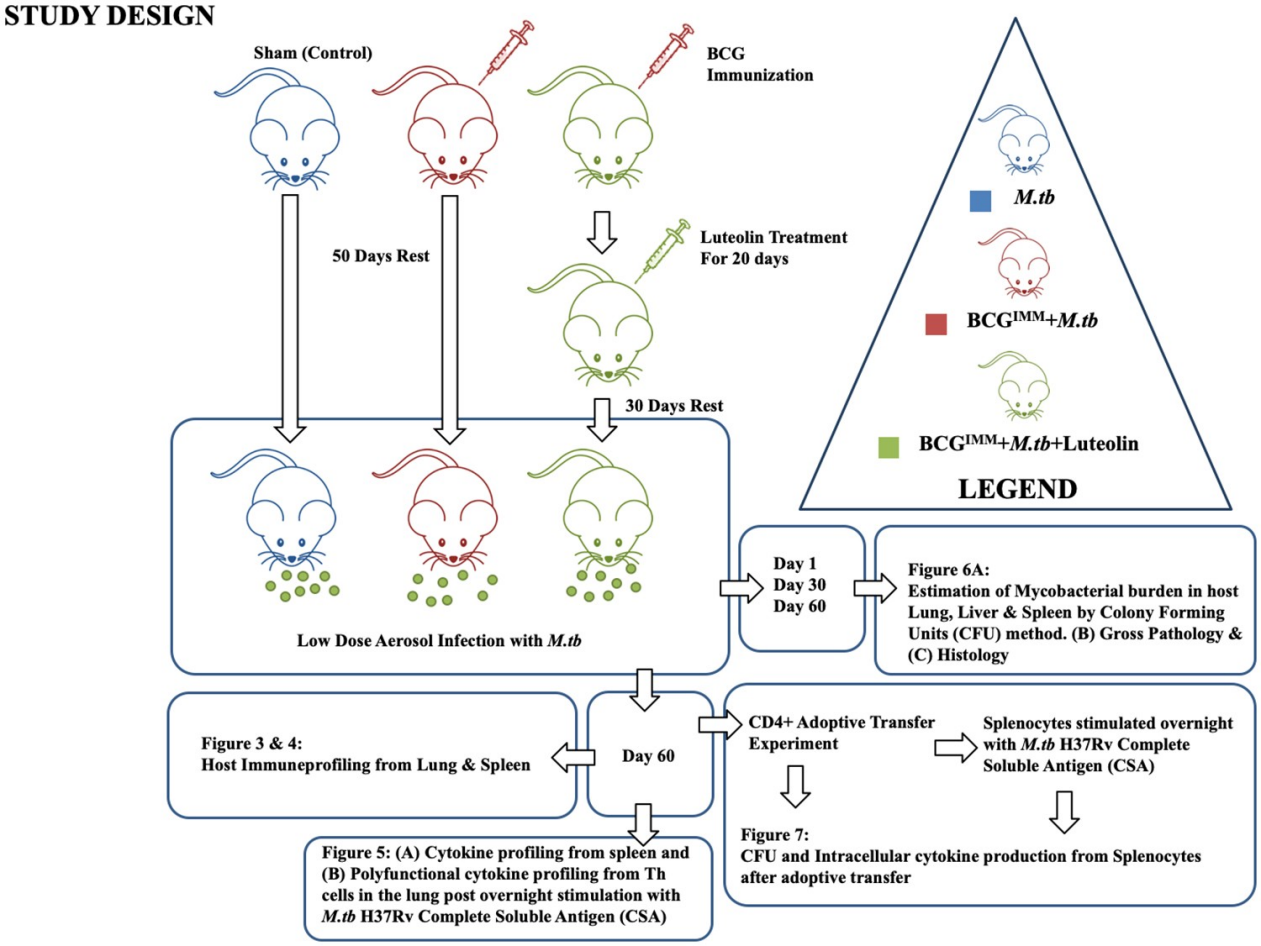
Study design.

**Fig 3:**
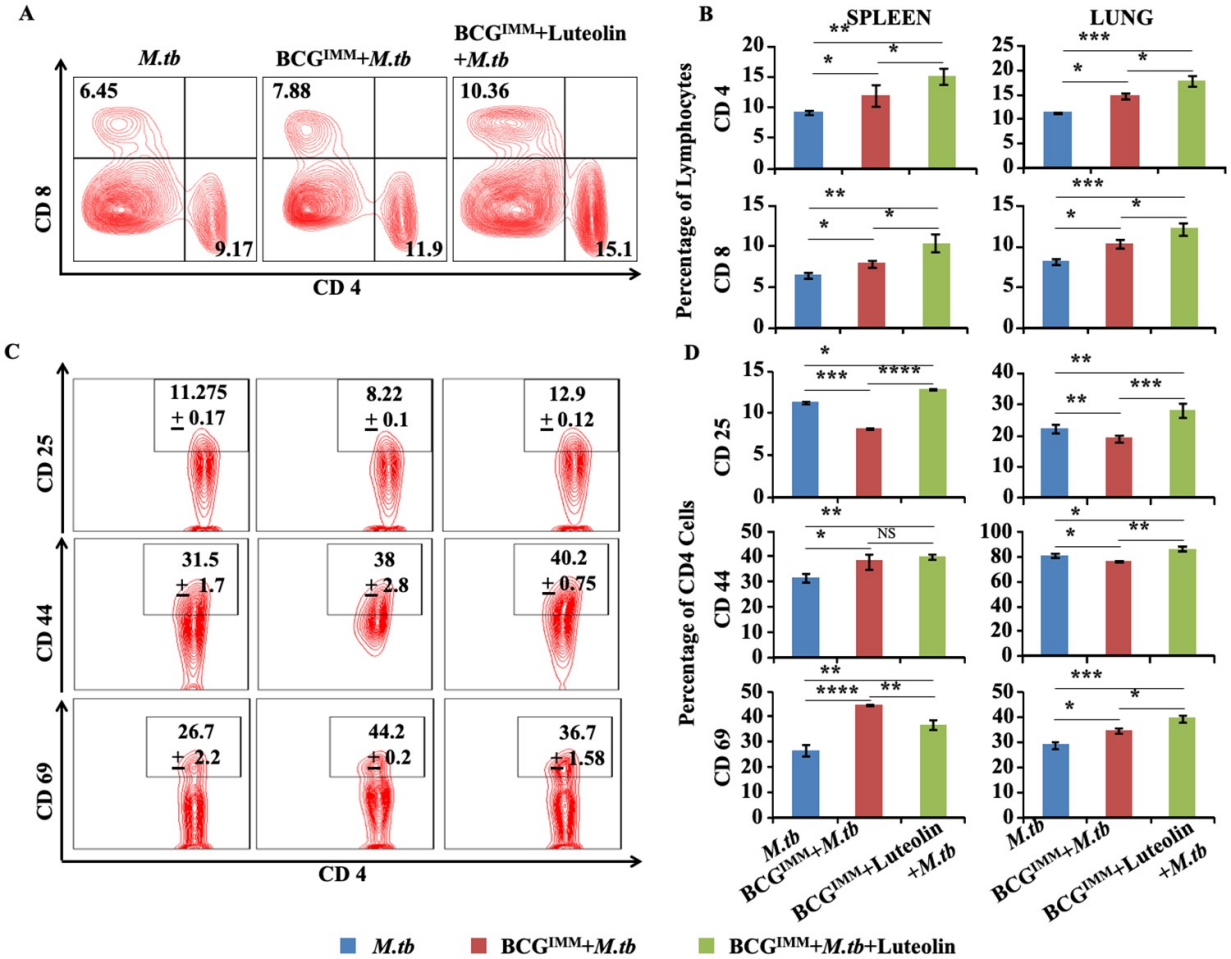
Luteolin promotes T cell responses in BCG-immunized mice. **(A)** Representative FACS plots and **(B)** combined data showing the proportion of CD4^+^ & CD8^+^ T lymphocytes in spleen and lung of mice of the indicated study groups at 60 days post-infection. **(C)** Representative FACS plots and **(D)** combined data showing activation profile of CD4^+^ T cells in spleen and lung of infected mice of the indicated study groups at 60 days post-infection. Data represented as the mean±STDEV values from data pooled from three experiments with five mice per experiment. Differences were considered significant at P<0.05 using one way ANOVA. *p<0.05, **p<0.005, ***p<0.0005.

To provide insight into the T cell response induced by luteolin in BCG-vaccinated mice, we analysed T lymphocytes from various experimental groups of mice: *M*.*tb* (sham-vaccinated, vehicle-treated followed by aerosol infection with *M*.*tb* strain **H37Rv**), **BCG**^**IMM**^**+*M*.*tb*** (BCG-vaccinated, vehicle-treated followed by aerosol infection) and **BCG**^**IMM**^**+Luteolin+*M*.*tb*** (BCG-vaccinated, luteolin-treated followed by aerosol infection) (Fig 2). Flow cytometric analysis (S3 Fig) revealed that CD4^+^ and CD8^+^ T cells in lungs and spleen of mice treated with luteolin were significantly increased as compared with mice immunized with BCG only (Fig 3A & 3B). Next, we determined the activation status of these cells by staining with anti-CD25, -CD44 and -CD69 antibodies. Fig 3C & 3D demonstrates that a large number of cells were indeed activated.

### Luteolin treatment during BCG vaccination promotes T_CM_ cells in mice challenged with *M*. *tuberculosis*

**Fig 4:**
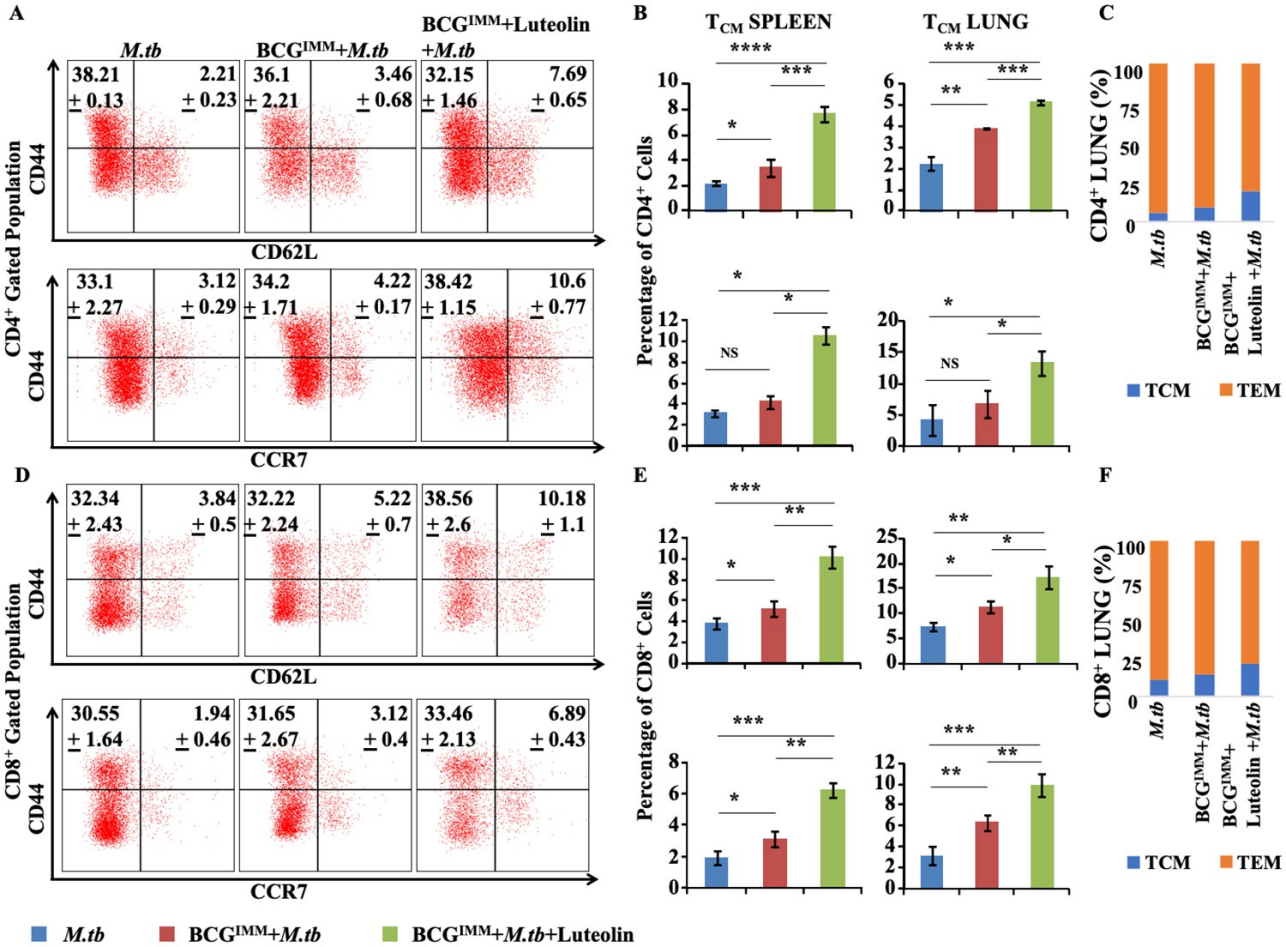
Luteolin induces increased T_CM_ responses in BCG-immunized mice. **(A&D)** Representative FACS plots, **(B, C, E and F)** combined data showing memory responses in the CD4^+^ T cell compartment **(A-C)** and CD8^+^ T cell compartment **(D-F)** in spleen and lung of infected mice of the indicated study groups at 60 days post-infection. Data represented as the mean±STDEV values from data pooled from three experiments with five mice per experiment. Differences were considered significant at P<0.05 using one way ANOVA. *p<0.05, **p<0.005, ***p<0.0005.

**Fig 5:**
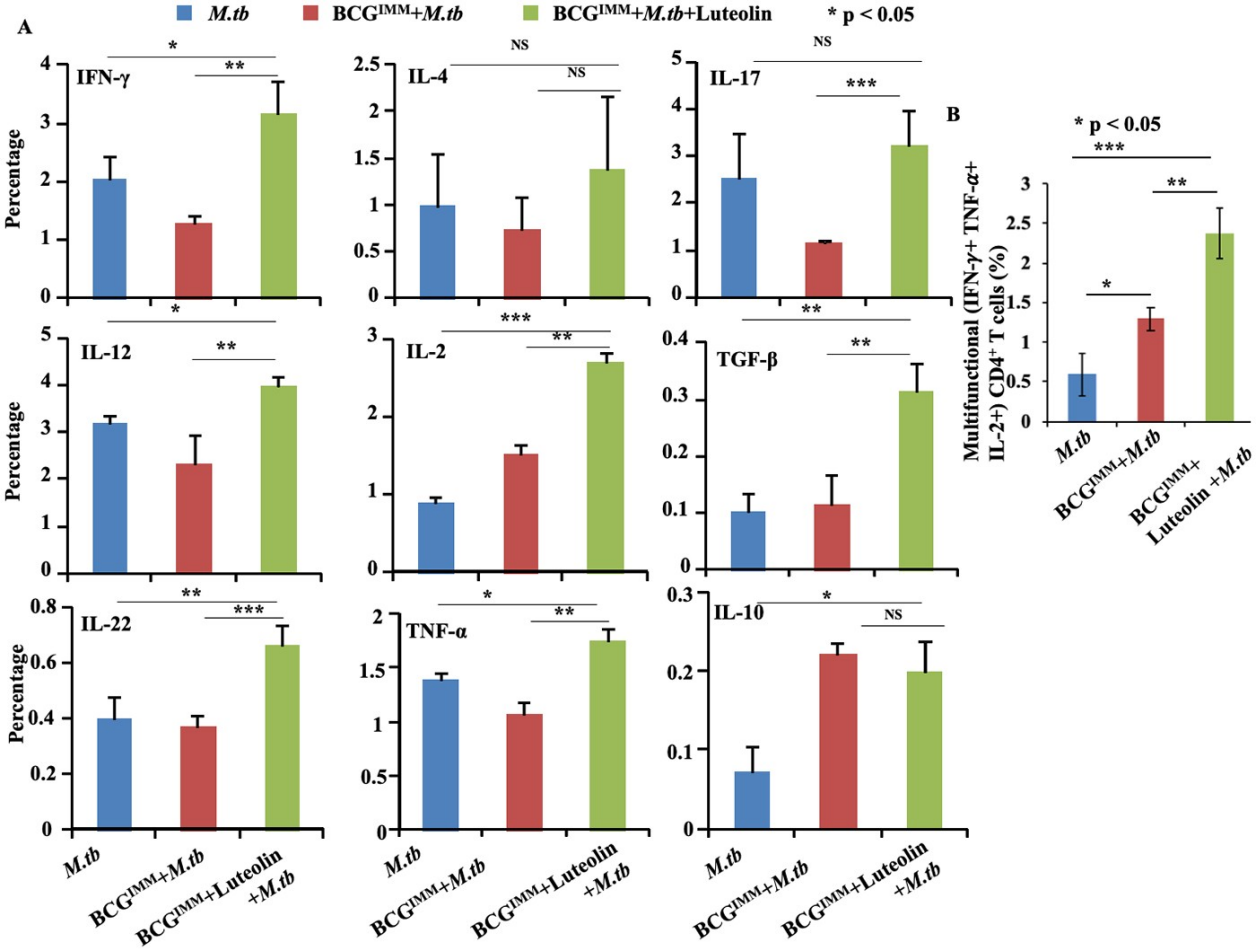
*M*.*tb* antigen-specific cytokine response. **(A)** Splenocytes from the indicated groups of mice were stimulated with *M*.*tb* H37Rv complete soluble antigen *ex vivo*, and the percentages of splenocytes producing cytokines were measured with flow cytometry. **(B)** Polyfunctional cytokine profiling from Th cells in the lung of the mice. Data represented as the mean±STDEV values from data pooled from three experiments (A) and two experiments (B) with five mice per experiment. Differences were considered significant at P<0.05 using one way ANOVA. *p<0.05, **p<0.005, ***p<0.0005.

Long term immunity is mostly dependent on the total pool of T_CM_ cells. However, BCG primarily induces T_EM_ cells in the lung, which might contribute to its limited vaccine efficacy [23]. We determined the generation of T_CM_ cells in immunized and luteolin-treated mice (Fig 4). Phenotypic analyses revealed that CD44^hi^CD62L^hi^ and CD44^hi^CCR7^hi^ T_CM_ cells in both CD4^+^ (Fig 4A–4C) and CD8^+^ (Fig 4D–4F) compartments were significantly higher in the luteolin-treated mice than in any of the other experimental groups post infection. However, T cells in all experimental groups exhibited comparable memory phenotype with a stronger antigen-specific Th1 response in the luteolin treatment group before infection (S4 Fig). The nature of the effector memory response in these experimental groups correlated well with their bacterial burden. As we and others have previously reported that efficient pathogen clearance is attained by Th1 and Th17 cell responses [24,25], we next determined the production of representative cytokines by these cells using intracellular staining. We found that luteolin-treated mice generated significantly increased levels of IFN-γ- and IL-17-producing Th cells, but we found no significant differences in IL-4-producing cells (Fig 5A). IL-22 production was also found to be higher in the luteolin-treated group of mice (Fig 5A). These observations suggested that luteolin enhances BCG vaccine efficacy by increasing the T_CM_:T_EM_ ratio of memory T cells with either a Th1 or Th17 phenotype as well as by induction of IL-22 (S5 Fig). Moreover, we also observed stronger polyfunctional Th cell responses (Fig 5B) in the lung of luteolin-treated mice (S6A & S6B Fig).

### Luteolin treatment enhances BCG vaccine-induced host protection against TB

**Fig 6:**
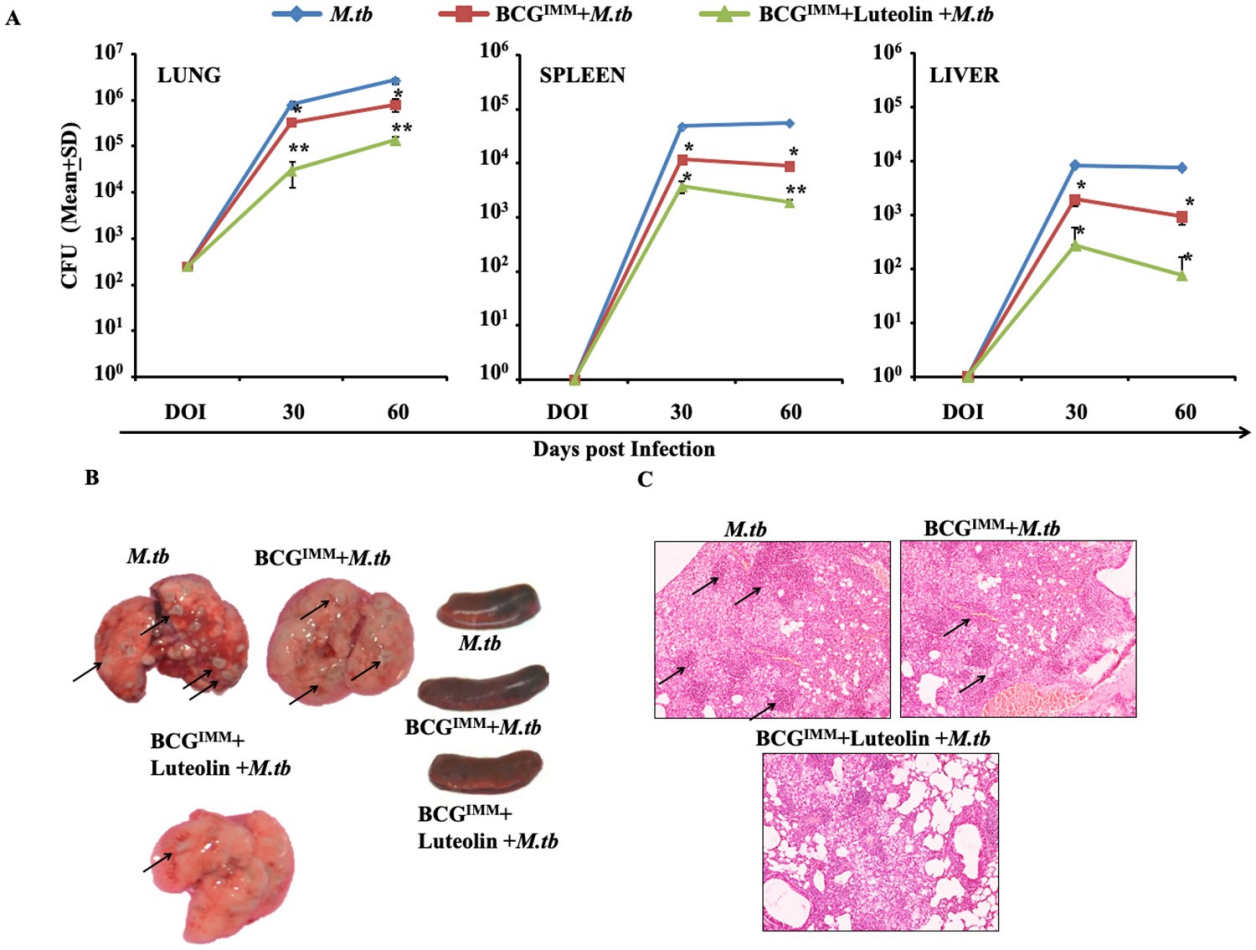
Luteolin treatment of BCG-immunized mice confers improved protection against TB. Mice immunized with BCG were treated with 5 mg/kg of luteolin or vehicle for 20 days and rested for an additional 30 days. These immunized mice and a control group of non-immunized mice were then infected with *M*.*tb*. Three randomly selected mice from various groups were euthanized at the indicated time points and lung, liver and spleen were harvested and homogenised. **(A)** CFUs from the lung, spleen and liver homogenates of *M*.*tb* infected mice. **(B)** Gross pathology of lungs and spleen at different time points after *M*.*tb* infection. Arrows indicate granulomatous lesions. **(C)** Lungs were dissected out and preserved in 10% neutral buffered Formalin (10% NBF). These preserved lungs were then processed for paraffin embedding, sectioning and staining with Hematoxylin and Eosin (H&E). Arrows indicate granulomatous lesions. Data represented as the mean±STDEV values from data pooled from three experiments with five mice per experiment. Differences were considered significant at P<0.05 using one way ANOVA. *p<0.05, **p<0.005, ***p<0.0005.

Prior studies have shown that luteolin demonstrates its immunomodulatory activities by inhibiting Kv1.3 [17,26]. It was recently shown that functional blockade of the Kv1.3 channel can enhance antimycobacterial immunity by promoting T_CM_ responses [15,27]. Therefore, these observations prompted us to test the effects of luteolin on BCG-induced vaccine efficacy. For this purpose, we treated BCG-immunized mice with luteolin at 5 mg/kg body weight starting 1 day after immunization, for a period of 20 days. These mice were then rested for 1 month and challenged with *M*.*tb* strain H37Rv. *M*.*tb* pathogenic burden was estimated by Colony Forming Unit (CFU) determination in lung, spleen and liver at various time points. Luteolin-treated mice displayed increased host protection, as revealed by reduced bacterial burden in various organs (Fig 6A), and reduced macroscopic, granulomatous lesions in lung and spleen (Fig 6B). This was further confirmed by histological analyses, which revealed reduced numbers of granulomas (Fig 6C).

### T cells from luteolin-treated, BCG-immunized mice confer improved protection against TB upon adoptive transfer

**Fig 7:**
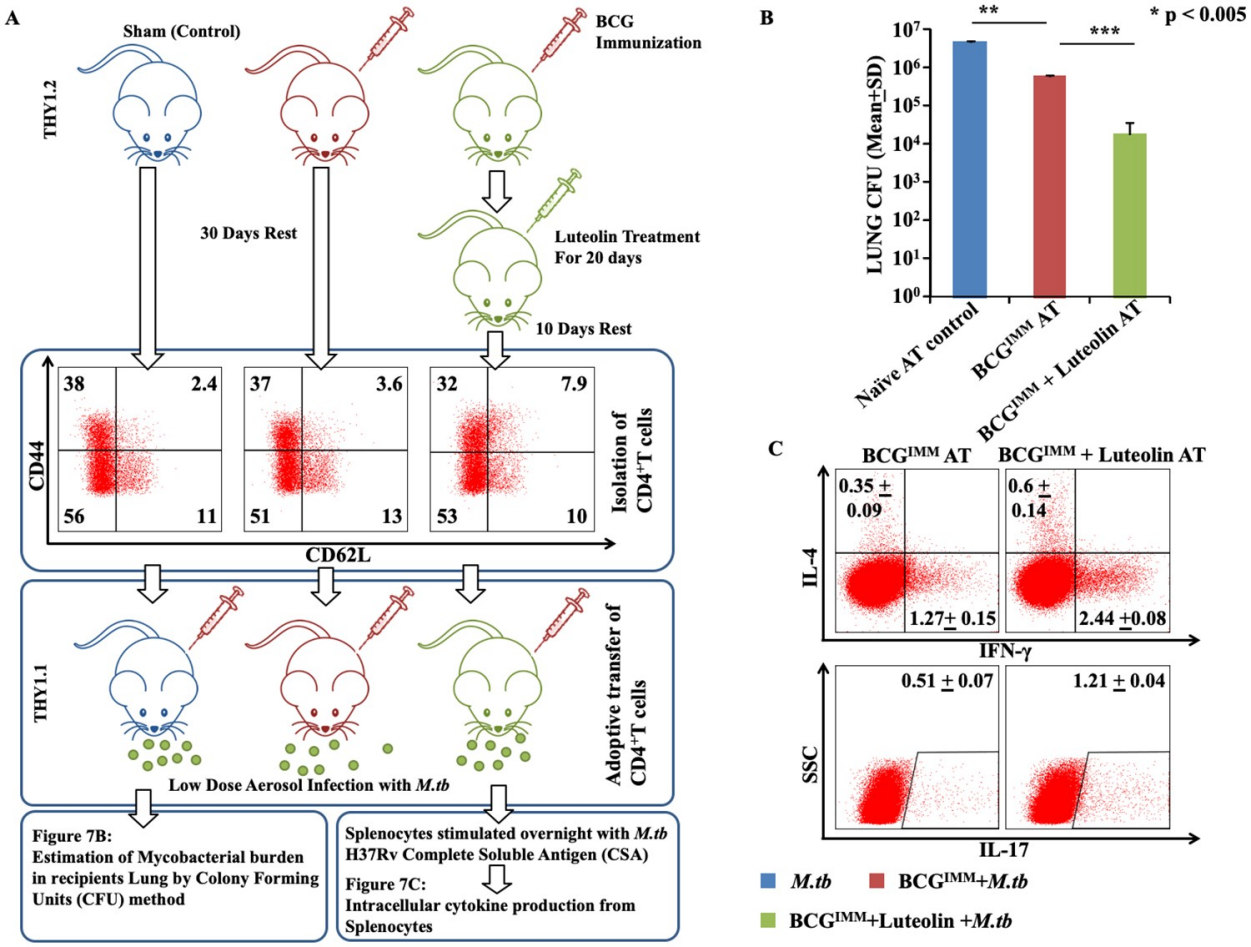
Adoptively transferred T cells from luteolin-treated and BCG-immunized mice confer improved protection against TB. **(A)** Experimental Layout: CD4^+^ T cells were isolated from congenic wild-type Thy1.2 mice immunized with BCG followed by luteolin treatment for 20 days and rested for 10 days. CD4^+^ T cells (10x10^6^) were adoptively transferred into γ-irradiated (sub-lethal dose of 800 rads/mice) Thy1.1 congenic mice followed by infection with *M*.*tb*. Twenty days after infection, spleen and lungs were isolated. (B) CFUs were estimated from lung homogenates of the different groups. (C) Splenocytes were challenged with *M*.*tb* complete soluble antigen ex vivo. T cells were then stained for the intracellular cytokines IL-4 vs. IFN-γ and IL-17. Data represented as the mean±STDEV values. Differences were considered significant at P<0.05 using one way ANOVA. Out of twelve mice within each group, only 5 mice in the BCG immunized control group and 6 mice in the luteolin control group survived on day 20. *p<0.05, **p<0.005, ***p<0.0005.

In order to accurately assess the role of luteolin-induced T_CM_ enrichment in boosting BCG-induced immunity and to rule out the other reported immunostimulatory functions of luteolin [19,28], we performed T cell adoptive transfer experiments. Congenic wild-type Thy1.2^+^ mice were immunized with BCG followed by luteolin treatment (5 mg/kg) for 20 days and then rested for 10 days. CD4^+^ T cells (10x10^6^) were adoptively transferred into γ-irradiated (sub-lethal dose of 800 rads/mice) Thy1.1^+^ congenic mice followed by infection with a low-dose aerosol challenge of *M*.*tb* strain H37Rv. Twenty days after infection, spleen and lungs were isolated from the surviving mice in each group for CFU estimation (Fig 7A & 7B) and antigen-specific intracellular cytokine responses (Fig 7A & 7C). Results showed that *surviving* recipient mice receiving T cells from luteolin-treated mice exhibited reduced CFUs (Fig 7B) and increased IFN-γ- and IL-17-producing, donor-derived (Thy1.2^+^) CD4^+^ T cells (Fig 7C). Since the cause of mortality in the adoptively transferred mice could not be objectively attributed to radiation toxicity or the infection, only mice surviving at the end of the study were used for CFU estimation and assessing T cell responses.

## Discussion

Successful vaccine efficacy against *M*.*tb* infection is predominantly dependent on the induction of memory cells with a central memory phenotype (CD62L^hi^CD44^hi^) [2,10,11,24,29–31], whereas induction of cells with an effector memory phenotype (CD62L^lo^CD44^hi^) is associated with active disease [32–34]. In this context, it has been shown that recombinant BCG vaccines with increased capacity to induce T_CM_ cells exhibit increased vaccine efficacy [11,35]. Several recombinant BCG vaccines with this property are in the development and testing pipeline [36–39].

Naïve T (CCR7^+^CD45RA^+^) and T_CM_ (CCR7^+^CD45RA^–^) cells require antigen priming in lymph nodes before they migrate to inflammatory sites, whereas terminally differentiated T_EM_ cells (CCR7^–^CD45RA^–^) rapidly enter inflamed tissues, produce copious amounts of cytokines, and exhibit immediate effector function [40,41]. Terminally differentiated human CCR7^–^CD45RA^−^T_EM_ cells have been reported to strongly upregulate Kv1.3 K^+^ ion channel expression. Resting (unstimulated) T cells of all subsets express about 200–400 Kv1.3 channels per cell along with very few calcium-activated K^+^ (IKCa1) channels. After activation, naïve T and T_CM_ cells upregulate IKCa1 (500–600 IKCa1 channels expressed per cell), whereas T_EM_ cells upregulate Kv1.3 (1,500–1,800 Kv1.3 channels expressed per cell) [42]. In fact, the Kv1.3^high^IKCa1^low^ phenotype has been suggested as a specific functional marker for activated T_EM_ cell subsets belonging to both the CD4^+^ and CD8^+^ compartments [42]. Since T_EM_ cells expressed significantly higher Kv1.3 levels and lower IKCa1 levels than naïve T and T_CM_ cells post-activation, selective Kv1.3 channel blockade suppresses proliferation of T_EM_ cells without affecting naïve T or T_CM_ cells [42]. Selective inhibition of Kv1.3 channels therefore appears to be a potential approach for generating T_CM_-driven long-term protective immunity against TB. While robust and rapid recall responses critically rely on the maintenance of memory T cells, this may also have detrimental effects such as depletion of the T_CM_ pool due to recurrent clonal expansion of T_EM_ cells during persistent infection by the same pathogen or due to recurrent exposure to related non-pathogenic species or strains [15,27]. The critical role of Kv1.3 in effector T cell function has already been explored as a target to suppress effector T cell functions in severe inflammatory diseases [26,43–45]. Kv1.3 loss of function in mice caused a delay in T_CM_ to T_EM_ progression by inhibiting cell cycle progression at the G2/M stage. This was mediated by up-regulation of SMAD3 phosphorylation, accelerating its translocation to the nucleus, where SMAD3 binds with the p21^cip1^ promoter and subsequently suppresses expression of the cell cycle genes cyclin-dependent kinase (Cdk)1 and cyclin B1 [13]. Nevertheless, Kv1.3-deficient mice were otherwise healthy and developed a normal immune system, with similar proportions of T lymphocytes in the spleen and thymus and similar proliferative responses of splenocytes to challenge with Concanavalin A or anti-CD3 antibodies when compared to wild type control mice [46]. A recent study reported that blockade of Kv1.3 by addition of clofazimine during immunization of mice with BCG enhances the available pool of T_CM_ cells, which provides superior protection against pulmonary TB [15]. Clofazimine, despite being a very successful antileprosy drug, has some limitations for use in a vaccine setting, more specifically in infants. First of all, it is a class 5 reserved drug for TB and is currently only being used in long treatment regimens of extremely drug-resistant (XDR) TB. Further, the half-life of clofazimine in lungs is estimated at over 4 weeks, and it accumulates in various organs [16]. Employing clofazimine during immunization of infants and adults appears suboptimal. Therefore, in the current study we interrogated the efficacy of a biologically safe alternative Kv1.3 inhibitor, luteolin, which is gaining popularity as a food supplement and is available in various pediatric and adult formulations [47]. Our study shows that luteolin treatment during BCG vaccination effectively alters the T_CM_:T_EM_ cell ratios, and demonstrates improved vaccine efficacy against *M*.*tb* infection compared with BCG alone. Luteolin generated an expanded T_CM_ pool that dictated efficient recall responses, which are considered imperative for vaccine efficacy and longevity of protective immune responses. T_CM_ cells are believed to be a perpetual source of T_EM_ cells, which are responsible for protection from infections by induction of a rapid effector response. Our adoptive transfer experiments clearly demonstrated the memory T cell-mediated protection and that these memory cells exhibit Th1 and Th17 phenotypes and are host-protective (Fig 7).

In summary, we have shown that luteolin alters the T_CM_:T_EM_ cell ratio when used during BCG vaccination and thus exhibits improved vaccine efficacy by inducing enhanced T_CM_ responses and augmenting Th1 and Th17 responses. Whether luteolin can improve disease protection in individuals already immunized with BCG will be a topic for future investigation.

## Material and methods

### Ethics statement

Animal experiments performed were in accordance with the guidelines approved by the 53rd Meeting of the Institutional Animals Ethics Committee held on 11^th^ February, 2014 at International Centre for Genetic Engineering and Biotechnology (ICGEB) (Approval ID: ICGEB/AH/2014/01/RGP-13), New Delhi, India as well as guidelines issued by the Department of Biotechnology (DBT), Government of India. All mice used for experiments were ethically sacrificed by asphyxiation in carbon dioxide according to institutional and DBT regulations.

### Mice

C57BL/6 mice that were Thy1.1^+^ or Thy1.2^+^ were initially purchased from The Jackson Laboratories (Bar Harbor, ME) and thereafter maintained at our specific pathogen-free animal facility at ICGEB. Mice used for infections were housed under barrier conditions in the Tuberculosis Aerosol Challenge Facility (TACF) of ICGEB and treated humanely as per the specified Animal Care protocols.

### *In vitro* T cell stimulation

Splenocytes isolated from naïve C57BL/6 mice were enriched for T cells by nylon wool method [48] followed by 6 hours of rest to bring the cellular activity to basal levels. They were then stimulated with plate-bound α-CD3 (1 μg/ml) and soluble α-CD28 (2 μg/ml) antibody in the presence or absence (control) of luteolin for 48 hours. These cells were then collected and surface-stained for FACS analysis.

### *M*.*tb* low-dose aerosol infection of mice

*M*.*tb* strain H37Rv (ATCC 27294; American Type Culture Collection, Rockville, MD) was a kind gift from the Colorado State University repository (Fort Collins, CO). Mouse infections were performed in accordance to the low-dose aerosol infection model using a Madison Aerosol Chamber (University of Wisconsin, Madison, WI) with the nebulizer pre-calibrated at ~200 CFU/mice. *M*.*tb* strain H37Rv was grown to mid-log phase (OD_600_ ∼0.6) in Middlebrook 7H9 media (Difco, USA) with 0.1% Tween 80 (Sigma, USA), 0.2% glycerol and 10% Middlebrook albumin, dextrose and catalase (ADC) enrichment medium (Difco, USA). Bacteria were stored at −80°C in 20% glycerol stocks for further experiments. For aerosol infection, cultures were washed twice with PBS and made into a single cell suspension by passing through a 26-gauge syringe ten times followed by two passes through a 30-gauge syringe. Fifteen ml of the *M*.*tb* strain H37Rv single cell suspension (20X10^6^ cells per ml) was placed in the Nebulizer reservoir of the Madison Aerosol Chamber calibrated to deliver the desired CFUs of bacteria into the lungs of mice kept in the chamber in 15 minute cycles. At 24 hours after aerosol challenge 3 mice were euthanized for quantification of pathogen delivery to lungs by measuring CFUs in lung homogenates. Mice were found to be infected with ∼220 CFU of *M*.*tb* strain H37Rv in their lungs. The mice were maintained under BSL-3 containment thereafter.

### Drug administration

Five mg/kg of luteolin (Sigma, USA) in 100 μl of PBS containing 5% DMSO v/v was administered intraperitoneally every day during the entire treatment phase, whereas controls were given vehicle only.

### Quantification of pathogen burden by Colony Forming Units (CFU)

Randomly selected mice were sacrificed at different time points, organs were harvested, homogenized in 0.2 μm filtered PBS containing 0.05% Tween 80 and plated onto 7H11 Middlebrook plates containing 10% OADC supplement. One hundred-fold, one thousand-fold, and ten thousand-fold diluted lung cell homogenate and ten-fold and one hundred-fold diluted spleen and liver cell homogenates were plated in doublet on 7H11 plates and incubated at 37°C for ~21 days. CFUs were counted and pathogen burden in lung, liver and spleen was estimated.

### Flow cytometry: Surface and intracellular staining

Spleens and lungs were isolated from mice and macerated by frosted slides in ice cold RPMI 1640 (Gibco, Invitrogen, UK) containing 10% FBS to prepare a single cell suspension. Red blood cells (RBCs) were lysed with RBC cell lysis buffer, incubated at room temperature for 3–5 minutes and washed with 10% RPMI 1640. The cells were counted and 1×10^6^ cells were used for surface staining. For intracellular staining 1×10^6^ cells were cultured per well in 12-well plates (Nunc, USA) and activated with *M*.*tb* H37Rv Complete Soluble Antigen (CSA) overnight, and 10 μg/ml Brefeldin A (eBiosciences, USA) was added during the last 6 hours of culture. Cells were washed twice with PBS and stained with antibodies directed against surface markers. After staining, cells were washed again with PBS and fixed with 100 μl fixation buffer (eBiosciences, USA) for 30 minutes, then re-suspended in 200 μl permeabilization buffer (eBiosciences, USA) and stained with fluorescently labelled anti-cytokine antibodies. Fluorescence intensity of fluorochrome-labelled cells was measured by flow cytometry (FACS Canto II, BD Biosciences, USA). FACS Diva was used for acquiring the cells and final data analysis was performed by Flow Jo (Tree star, USA).

### Detection of cytokines

Cytokines in the serum of immunized, infected and infected and treated mice were assayed by Luminex microbead-based multiplexed assay using commercially available kits according to the manufacturer’s protocol (BioPlex, Bio-Rad, USA).

### Antigen-specific degranulation assay

Splenocytes isolated from randomly selected mice in different groups were isolated as described above and 2X10^6^ splenocytes per well were cultured in RPMI 1640 (Gibco, Invitrogen, UK) containing 10% FBS in a 12-well plate (Nunc, USA) at 37^°^C in 5% CO_2_ for 4 hours to bring the cellular activity to basal levels. Splenocytes were then challenged with 20 μg/ml of H37Rv CSA and cultured for an additional 2 hours, after which 5 μl Monensin (Golgi-Stop, BD Biosciences) and 5 μl of anti-CD107A-FITC antibody (BD Biosciences) were added per well and cultured for an additional 4 hours at 37°C in 5% CO_2_. These cells were then collected and surface-stained for FACS analysis.

### BCG immunization experiments

Mice were either sham vaccinated or immunized subcutaneously with 1×10^6^ CFUs of BCG in 100 μl of sterile saline as described [31]. After one day of rest these mice were treated with either vehicle (saline) or luteolin at 5 mg/kg of body weight every day for a total of 20 days. Mice were subsequently rested for 30 days. Mice were then challenged via the aerosol route with *M*.*tb* strain H37Rv as above and organs were harvested for determination of bacterial burden and immune-profiling at different time points.

### T Cell adoptive transfer experiments

For adoptive transfer experiments, Thy1.1^+^ mice were gamma-irradiated (4.6 rads/sec for 175 seconds) and rested for a day. CD4^+^ T cells were isolated using anti-CD4 beads over a magnetic column (MiltenyiBiotec, USA) from the lymph nodes of Thy1.2^+^ mice that were either naïve (Control) or had been previously immunized with BCG and treated with vehicle (BCG^IMM^ AT) or luteolin (BCG^IMM^ + Luteolin AT) for 20 days and then rested for an additional 10 days. These cells were then adoptively transferred into the irradiated recipient mice (10x10^6^ cells per mouse). After 4 days recipient mice were challenged with *M*.*tb* strain H37Rv through the aerosol route. Each group was comprised of 12 mice, of which 5 mice in the control group and BCG^IMM^ AT group and 6 mice in the BCG^IMM^+Luteolin AT group survived on day 20 post-infection. The surviving mice were euthanized for CFU estimation or immunological studies.

### Antibodies and reagents

We used the following antibodies: anti-CD3 (clone: 145-2C11)-PerCP-Cy5 or -APC, CD4 (clone: GK1.5, RM4-5)-FITC, -PerCP-Cy5 or -APC, CD8 (clone: 53–6.7)-FITC, -APC-H7, -PerCP-Cy5 or -APC, CD44 (clone: IM7)-APC, CD62L (clone: MEL-14)-PE, CD25 (clone: 3C7)-PE, -APC, FOXP3 (clone: MF23, R16-715)-APC, IFN-γ (clone: XMG1.2)-APC, IL-4 (clone: 11B11)-PE, IL-6 (clone: MPS-20F3)-PE, IL-10 (clone: JES5-16E3)-APC, IL-12 (clone: C15.6)-PE, IL-17 (clone: O79-289)-PE, IL-2 (clone: JES6-5H4)-PerCP or -FITC, IL-22 (clone: Poly5164)-PE, TNF-α (clone: MP6-XT22)-PE, TGF-β (clone: TW7-16B4)-APC (from Biolegend, USA), and CD69 (clone: H1.2F3)-PE (from eBiosciences, USA).

### Statistical analysis

Data are represented as mean±STDEV derived from at least three independent experiments. Statistical analyses were conducted using IBM SPSS20 software. Significant differences between the group means were determined by One way ANOVA followed by Post-hoc analysis with Tukey’s correction for Multiple comparison except for data depicted in S2 & S4 Figs where unpaired t test was peformed (SPSS software). A value of p<0.05 was considered statistically significant.

## Supporting information

S1 Fig In vitro bactericidal activity of luteolin (100 μM/mL). (TIF)

S2 Fig **(A)** Profiling of macrophage activation after luteolin treatment (25 μM/mL). **(B)** In vitro CFU assay in macrophages infected with *M*.*tb* and treated with luteolin.
(TIF)

S3 Fig Gating strategies employed for immune profiling of T cells. (TIF)

S4 Fig Bar diagram to show the percentage of different subsets of memory T cells in Lungs after immunization and luteolin treatment prior to infection. (TIF)

S5 Fig Cytokine profiling from splenocytes of the respective experimental groups. (TIF)

S6 Fig Gating strategies employed for the profiling of antigen-specific cytokine responses in T cells from splenocytes. (TIF)
