# Correction: Luteolin-mediated Kv1.3 K^+^ channel inhibition augments BCG vaccine efficacy against tuberculosis by promoting central memory T cell responses in mice

**DOI:** 10.1371/journal.ppat.1009896

**Published:** 2021-08-30

**Authors:** Dhiraj Kumar Singh, Ved Prakash Dwivedi, Shashi Prakash Singh, Anjna Kumari, Saurabh Kumar Sharma, Anand Ranganathan, Luc Van Kaer, Gobardhan Das

In Fig 6C, the incorrect images were used for the *M*.*tb*, BCG^IMM^+*M*.*tb* and BCG^IMM^+Luteolin+*M*.*tb* panels. Please see the correct Fig 6 here.

**Fig 6:**
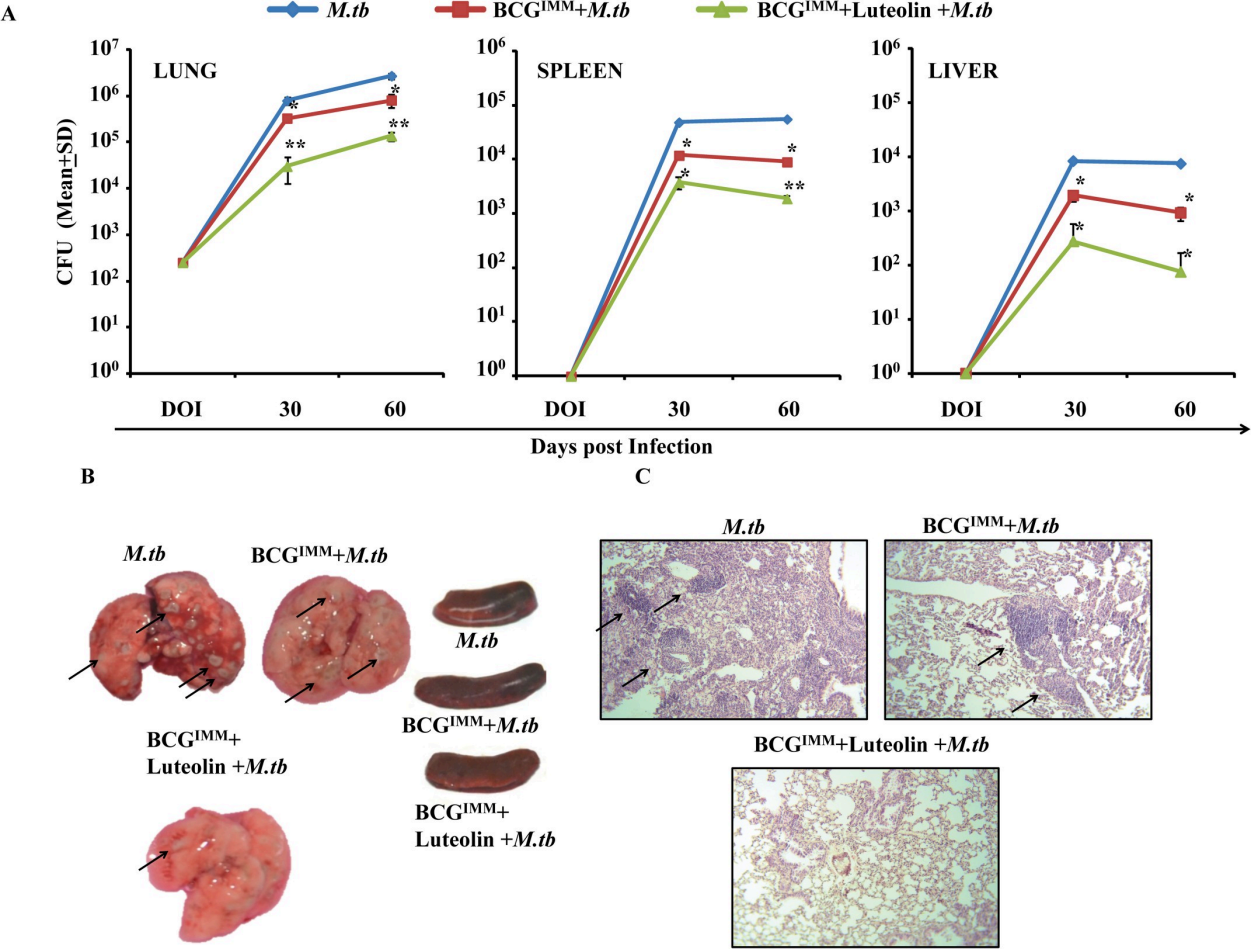
Luteolin treatment of BCG-immunized mice confers improved protection against TB. Mice immunized with BCG were treated with 5 mg/kg of luteolin or vehicle for 20 days and rested for an additional 30 days. These immunized mice and a control group of non-immunized mice were then infected with M.tb. Three randomly selected mice from various groups were euthanized at the indicated time points and lung, liver and spleen were harvested and homogenised. (A) CFUs from the lung, spleen and liver homogenates of M.tb infected mice. (B) Gross pathology of lungs and spleen at different time points after M.tb infection. Arrows indicate granulomatous lesions. (C) Lungs were dissected out and preserved in 10% neutral buffered Formalin (10% NBF). These preserved lungs were then processed for paraffin embedding, sectioning and staining with Hematoxylin and Eosin (H&E). Arrows indicate granulomatous lesions. Data represented as the mean±STDEV values from data pooled from three experiments with five mice per experiment. Differences were considered significant at P<0.05 using one way ANOVA. *p<0.05, **p<0.005, ***p<0.0005.

## References

[1] Singh DK, Dwivedi VP, Singh SP, Kumari A, Sharma SK, Ranganathan A, et al. (2020) Luteolin-mediated Kv1.3 K^+^ channel inhibition augments BCG vaccine efficacy against tuberculosis by promoting central memory T cell responses in mice. PLoS Pathog 16(9): e1008887. 10.1371/journal.ppat.1008887 32956412 PMC7529197

