# Supplementary figures and images for: Luteolin-mediated Kv1.3 K+ channel inhibition augments BCG vaccine efficacy against tuberculosis by promoting central memory T cell responses in mice
Source: PLoS Pathog. 2020 Sep 21;16(9):e1008887. doi: 10.1371/journal.ppat.1008887 (PMC7529197; doi:10.1371/journal.ppat.1008887)

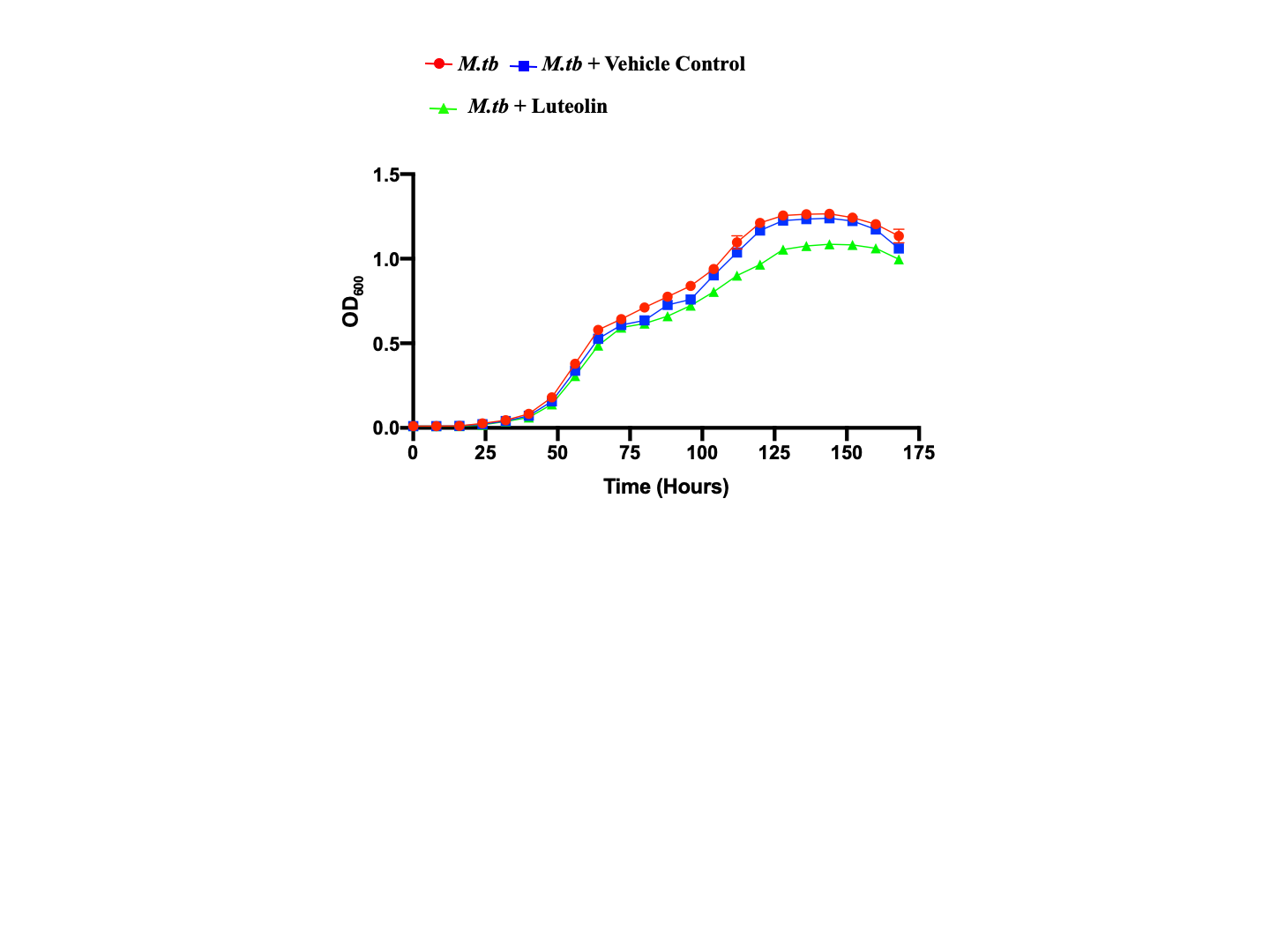

Supplement: S1 Fig — (TIF) [file ppat.1008887.s001.tif]

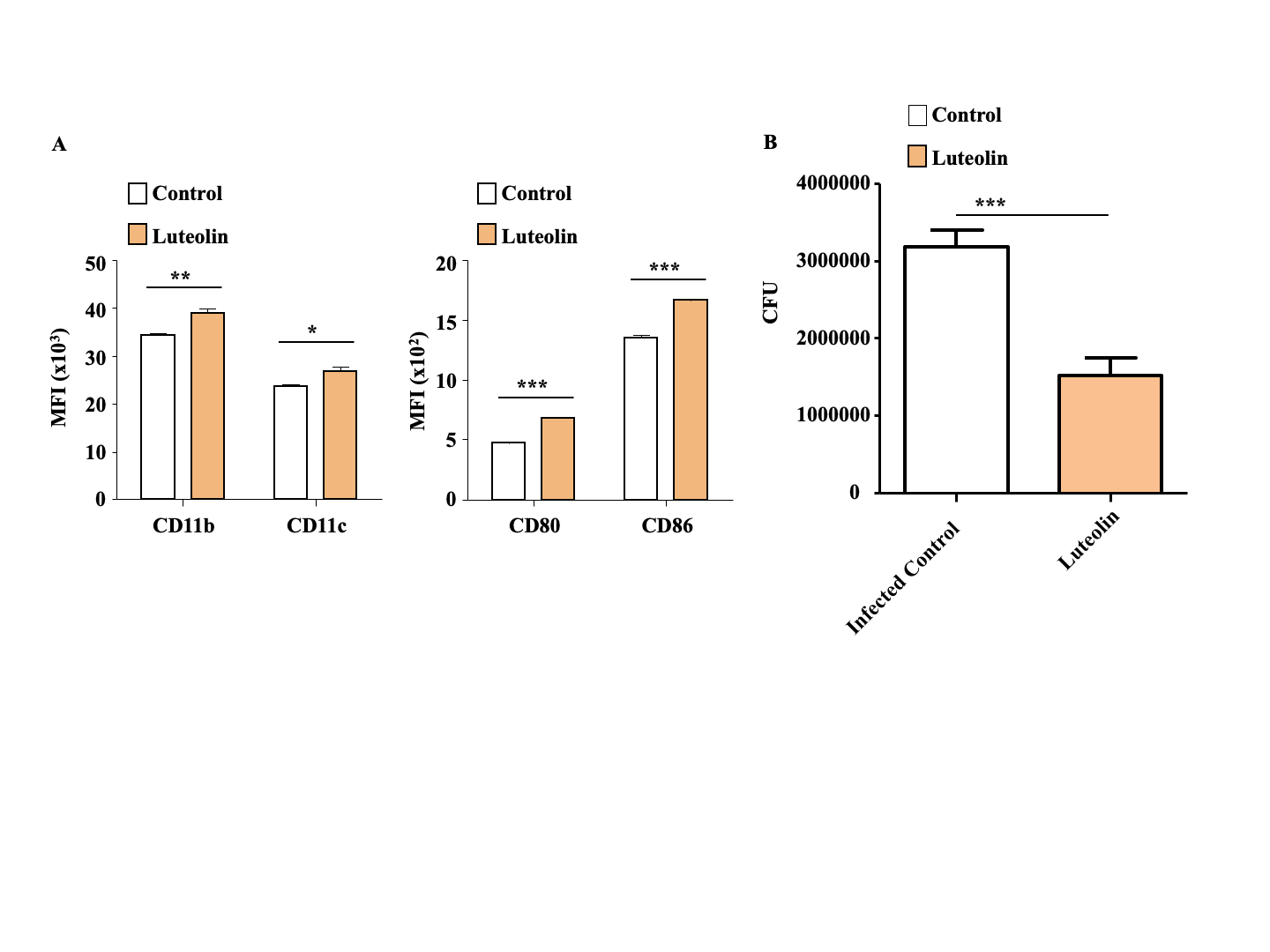

Supplement: S2 Fig — (A) Profiling of macrophage activation after luteolin treatment (25 μM/mL). (B) In vitro CFU assay in macrophages infected with M.tb and treated with luteolin. (TIF) [file ppat.1008887.s002.tif]

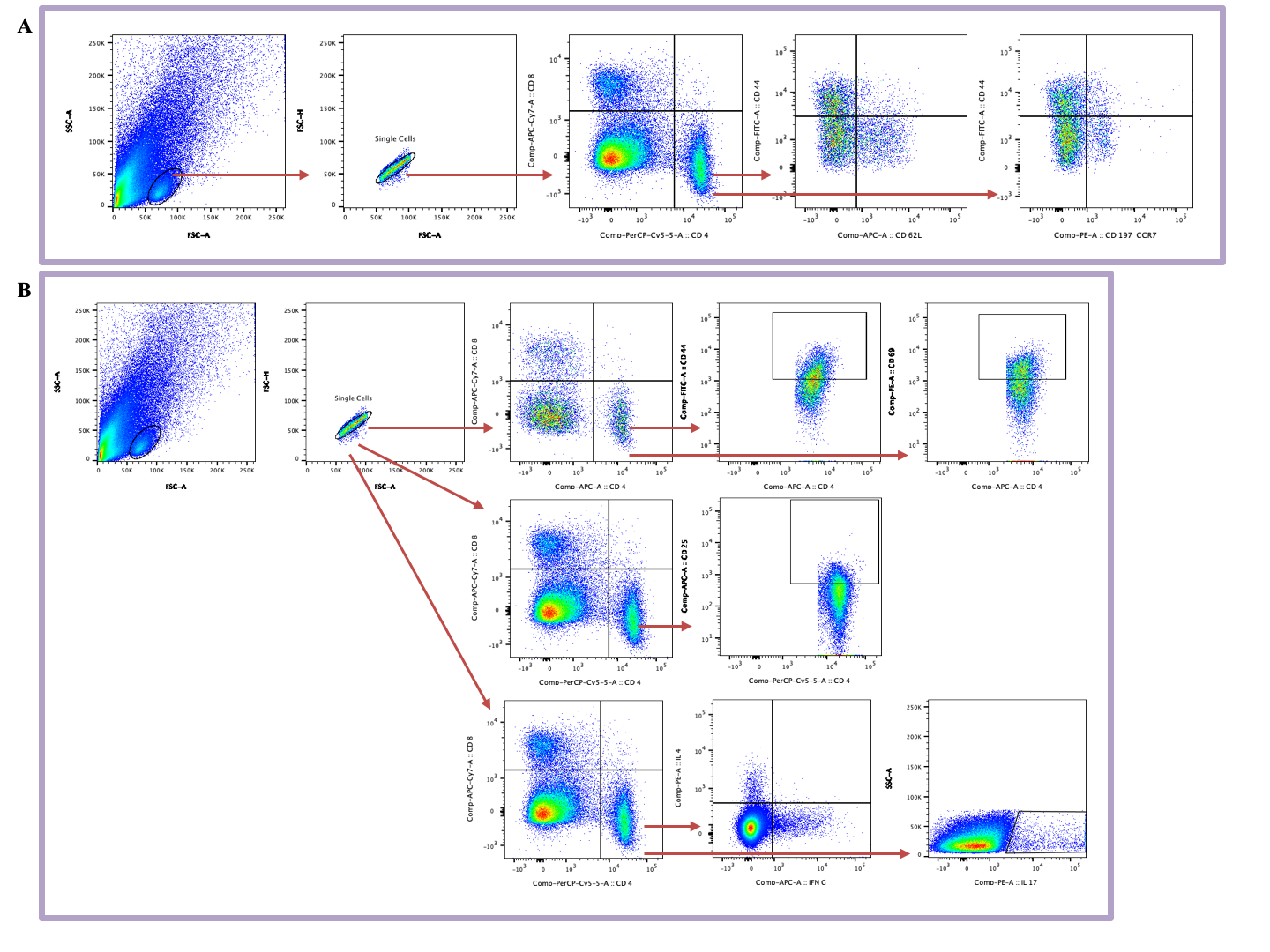

Supplement: S3 Fig — (TIF) [file ppat.1008887.s003.tif]

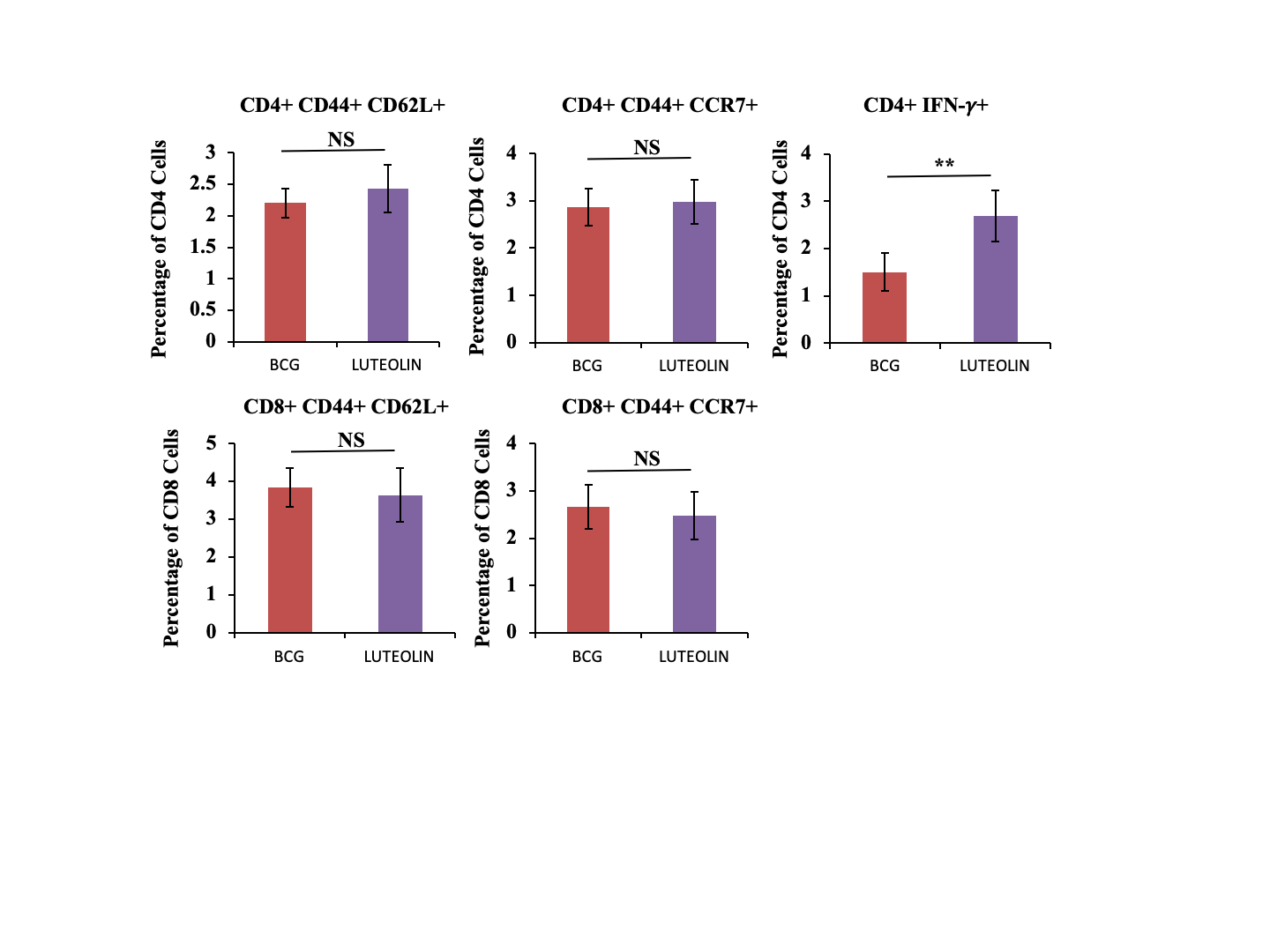

Supplement: S4 Fig — (TIF) [file ppat.1008887.s004.tif]

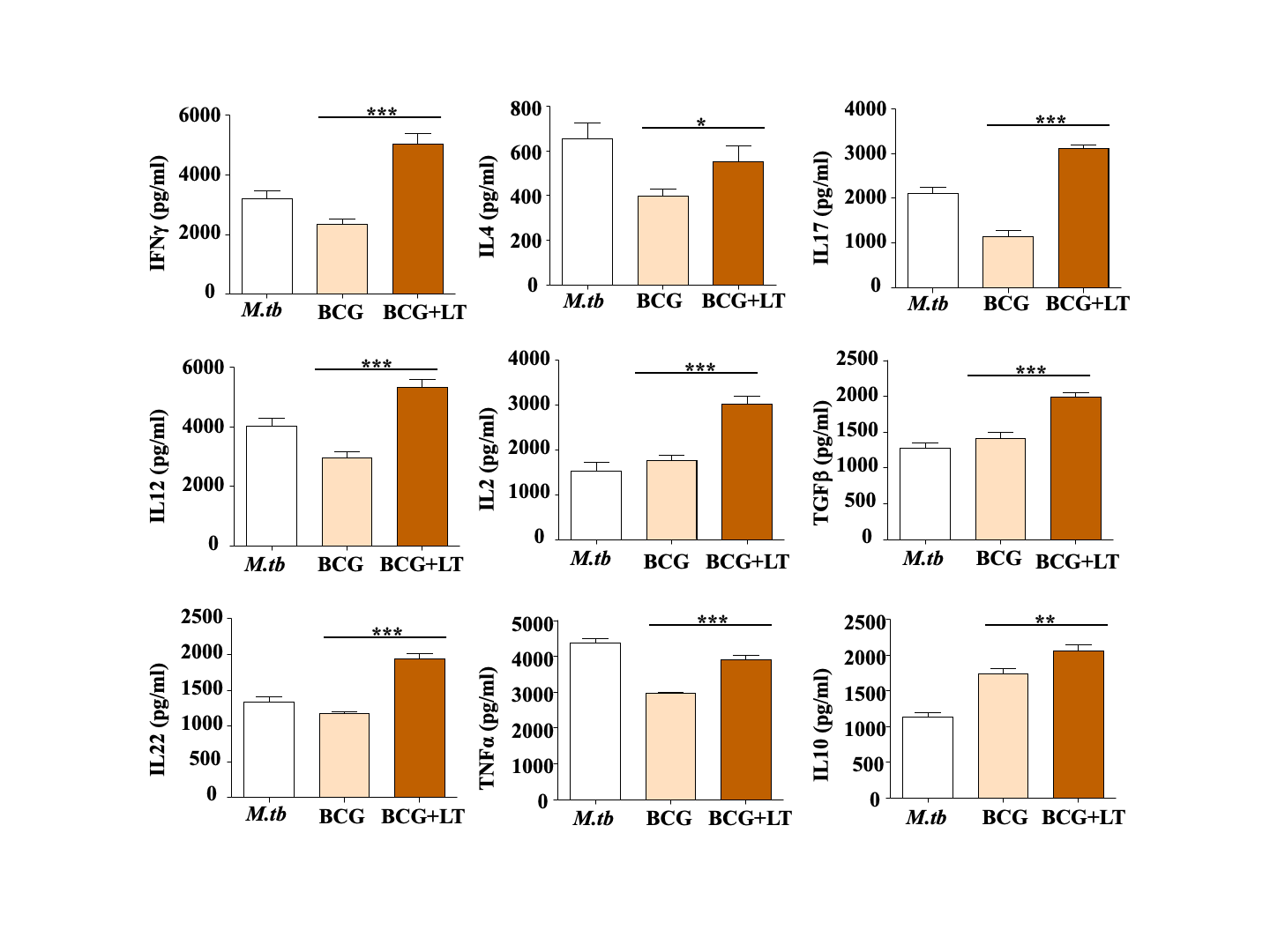

Supplement: S5 Fig — (TIF) [file ppat.1008887.s005.tif]

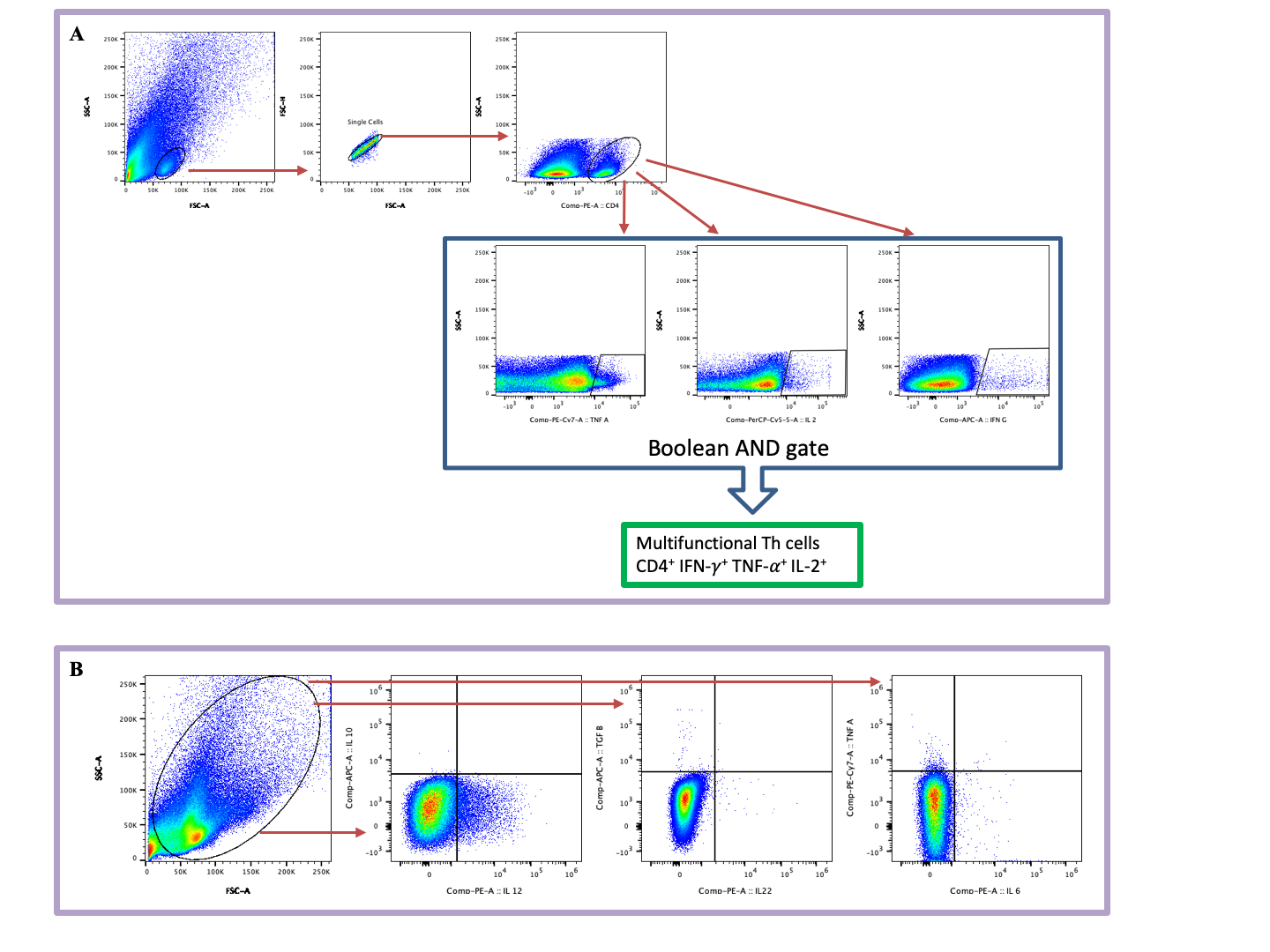

Supplement: S6 Fig — (TIF) [file ppat.1008887.s006.tif]
